# Model-based clustering of DNA methylation array data: a recursive-partitioning algorithm for high-dimensional data arising as a mixture of beta distributions

**DOI:** 10.1186/1471-2105-9-365

**Published:** 2008-09-09

**Authors:** E Andres Houseman, Brock C Christensen, Ru-Fang Yeh, Carmen J Marsit, Margaret R Karagas, Margaret Wrensch, Heather H Nelson, Joseph Wiemels, Shichun Zheng, John K Wiencke, Karl T Kelsey

**Affiliations:** 1 Department of Biostatistics, Harvard School of Public Health, Boston, Massachusetts, 02115, USA; 2 Department of Community Health, Center for Environmental Health and Technology, Brown University, Providence, Rhode Island, 02912, USA; 3 Department of Epidemiology and Biostatistics, University of California San Francisco, San Francisco, California, 94143, USA; 4 Department of Pathology and Laboratory Medicine, Brown University, Providence, Rhode Island, 02912, USA; 5 Department of Community and Family Medicine, Dartmouth-Hitchcock Medical Center, Lebanon, New Hampshire, 03756, USA; 6 Department of Neurological Surgery, University of California San Francisco, San Francisco, California, 94143, USA; 7 Division of Epidemiology and Community Health, University of Minnesota School of Public Health, Minneapolis, Minnesota, 55455, USA

## Abstract

**Background:**

Epigenetics is the study of heritable changes in gene function that cannot be explained by changes in DNA sequence. One of the most commonly studied epigenetic alterations is cytosine methylation, which is a well recognized mechanism of epigenetic gene silencing and often occurs at tumor suppressor gene loci in human cancer. Arrays are now being used to study DNA methylation at a large number of loci; for example, the Illumina GoldenGate platform assesses DNA methylation at 1505 loci associated with over 800 cancer-related genes. Model-based cluster analysis is often used to identify DNA methylation subgroups in data, but it is unclear how to cluster DNA methylation data from arrays in a scalable and reliable manner.

**Results:**

We propose a novel model-based recursive-partitioning algorithm to navigate clusters in a beta mixture model. We present simulations that show that the method is more reliable than competing nonparametric clustering approaches, and is at least as reliable as conventional mixture model methods. We also show that our proposed method is more computationally efficient than conventional mixture model approaches. We demonstrate our method on the normal tissue samples and show that the clusters are associated with tissue type as well as age.

**Conclusion:**

Our proposed recursively-partitioned mixture model is an effective and computationally efficient method for clustering DNA methylation data.

## Background

Epigenetics is the study of heritable changes in gene function that cannot be explained by changes in DNA sequence [1]. One of the most commonly studied epigenetic alterations is cytosine methylation, which occurs in the context of a CpG dinucleotide. Concentrations of CpGs known as CpG islands, when sufficiently methylated, are associated with transcriptional gene silencing tantamount to "one hit" as part of Knudson's two hit hypothesis of tumor suppressor gene inactivation [2]. DNA methylation associated gene silencing is a well recognized epigenetic mechanism that often occurs at tumor suppressor gene loci in human cancer. Hundreds of reports of methylation induced silencing at tumor suppressor genes in virtually all types of human cancer have been published [3,4].

While there has been a tremendous effort to characterize epigenetic alterations in cancer, surprisingly little work has been done in disease-free tissues. There is a basic need for epigenetic profiling of normal tissues to better understand the contribution of these profiles to tissue specificity, especially in the context of phenotypically important CpGs, where deregulation is associated with human diseases such as cancer. While efforts to characterize the methylation profiles of normal tissues in humans and mice have begun, and certain themes are slowly becoming apparent, relatively few reports have emerged [4-11]. Most CpGs or CpG regions have been found to have a bimodal distribution of methylation profiles, either hypo- or hypermethylated. Another theme is the disproportionate location of cell or tissue type dependent differentially methylated regions in non-CpG island sites [4,8]. Furthermore, the Human Epigenome Project showed that the human major histocompatability loci have differential methylation across various human tissues, but that differential methylation does not necessarily lead to differential expression [8]. It is therefore critical to first outline the basal-state of phenotypically important epigenetic marks that are known to contribute to cancer in order to have a background for comparison to other normal and diseased tissues. An approach which characterizes the basal epigenetic state is best suited to foster the discovery of epigenetic profiles that are associated with particular disease states, patient characteristics, exposures or other covariates that may contribute to pathogenesis.

Cluster analysis is often used to identify methylation subgroups in data [12,13] and, in particular, Siegmund et al. (2004) argue that model-based clustering techniques are often superior to nonparametric approaches [13]. Large-scale methylation arrays are now available for studying methylation genome-wide; the GoldenGate methylation platform from the manufacturer Illumina (San Diego, CA) simultaneously measures cytosine methylation at 1505 phenotypically-important loci associated with over 800 cancer-related genes. The result of the array is a sequence of "beta" values, one for each locus, calculated as the average of approximately 30 replicates (approximately 30 beads per site per sample) of the quantity max(*M*, 0)/(|*U*| + |*M*| + *Q*), where *U *is the fluorescent signal from an unmethylated allele on a single bead, *M *is that from a methylated allele, and *Q *is a constant chosen to ensure that the quantity is well-defined; an absolute value is used in the denominator of the formula to compensate for negative signals due to background subtraction. Note that each beta value is an approximately continuous variable lying between zero and one, where zero represents an unmethylated locus and one represents a methylated locus. As such, the beta value is appropriately modeled with a beta distribution; we further motivate the choice of beta distribution in the Methods section below. A data set consisting of such sequences produces a high-dimensional data-analysis problem which poses challenges for traditional clustering approaches. In addition, analysis of heterogeneous tissue data can lead to a large number of clusters, as we demonstrate below, which presents further challenges for clustering techniques. For example, nonparametric approaches rely on a choice of metric, which may be difficult to justify in the context of high dimensions and numerous clusters. On the other hand, in model-based clustering, multi-modality of the data likelihood may lead to numerical instability or difficulty in determining the best solution [14].

We propose a novel method for model-based clustering of data of the type produced by Illumina GoldenGate arrays. Our method makes use of a beta mixture model [15]. Although one could use BIC (or similar quantities) to select the number of clusters in the data set, we propose a recursive-partitioning algorithm that provides the number of clusters and a reliable solution in a shorter amount of time than sequential attempts with different numbers of assumed clusters. This is similar in spirit to the idea of recursive partitioning used in Hierarchical Ordered Partitioning and Collapsing Hybrid (HOPACH, [16]), in which clusters are recursively partitioned using a nonparametric algorithm such as PAM [17]. Our method is also an unsupervised variant of Hierarchical Mixtures of Experts [18], a fuzzy version of CART [19]. We also propose a method for reducing the number of loci considered in the analysis, and selecting the optimal number using an "augmented" BIC statistic. We also present a simulation study comparing its properties to those of competitor methods. Finally, we demonstrate the methodology on GoldenGate methylation array data obtained from 217 normal tissue samples.

## Results

Throughout the text, we assume a beta mixture model. For subject *i*, let **Y**_*i *_= (*Y*_*i*1_, ... *Y*_*iJ*_) be a vector of *J *continuous outcomes falling between 0 and 1, and let there be *n *such vectors. We posit a mixture model having *K *classes, so that subject *i *belongs to class *C*_*i *_∈ {1, ..., *K*} and conditional on class membership, each outcome *Y*_*ij *_is an independent Beta-distributed variable with parameters *α*_*kj *_and *β*_*kj *_depending on both class *k *and dimension *j*. The objective of our method is to obtain posterior class membership probabilities *w*_*ik *_= *P*(*C*_*i *_= *k *| **Y**_*i*_). Each class has a profile that is obtained from the collection of parameters *α*_*kj *_and *β*_*kj*_, or equivalently the first two moments:

(1) $$\begin{array}{l} \begin{array}{cc} E(Y_{ij}|C_{i}=k)=\mu_{jk}=\alpha_{kj}{\left(\alpha_{kj}+\beta_{kj}\right)}^{-1} & \text{and} \end{array}\\ \mathrm{var}(Y_{ij}|C_{i}=k)=\mu_{jk}(1-\mu_{jk}){\left(\alpha_{kj}+\beta_{kj}+1\right)}^{-1}. \end{array}$$

### Simulation

Table 1 displays the classification error and computation time resulting from a simulation study described below in the Methods section. In both cases simulated, the mixture models outperformed the nonparametric methods in terms of classification error. For Case I, based on normal tissue data and described below, our proposed recursive-partitioned mixture model outperformed all other methods, including the standard sequentially-fit mixture models where different values of *K *are assumed and the results compared via Bayesian Information Criterion (BIC). In addition, two different implementations of our method, each using different BIC criteria for splitting, produced identical results, including classification. For Case II, based on artificial parameters representing extremes of mean and variability, all mixture models performed equally well, and both versions of our proposed method produced the same results. In general, the mixture models had longer computation time than the nonparametric methods (hierarchical clustering and HOPACH); however, we note that the mixture models were implemented as interpreted code in R, while the nonparametric methods were precompiled programs with R interfaces. Note that the recursively-partitioned mixture model was anywhere from 3 to 8 times faster than the sequentially fit mixture model. Table 2 summarizes the number of classes found with hierarchical clustering in combination with dynamic tree cutting, with both versions of HOPACH, and with our proposed method using the standard BIC. For the cases considered, the mixture models almost always found the correct number of classes if *J *was high enough, while the nonparametric methods often had difficulty finding the correct number of classes. We obtained qualitatively similar results in additional simulations employing different distribution assumptions, though we omit the results.

**Table 1:** Classification error and computation time for various clustering methods applied to simulated data.

**Classification Error (%)**								
	***J***	**HC**	**DynTree**	**HOPACH (best)**	**HOPACH (greedy)**	**MM(1–6)**	**RPMM (ICL-BIC)**	**RPMM (BIC)**
**Case 1**	25	33.2	44.7	9.9	16.4	12.6	15.5	15.4
	50	32.5	43.8	5.0	10.0	6.2	5.5	5.5
	500	33.9	38.4	3.5	11.3	1.5	0.1	0.1
	1000	34.0	38.5	9.2	14.4	1.1	0.1	0.1
								
**Case 2**	5	59.4	60.5	65.1	65.8	59.4	59.4	59.4
	10	58.9	60.0	66.9	67.5	59.2	59.2	59.2
	25	30.0	39.6	4.1	8.1	0.0	0.0	0.0
	50	29.9	39.6	3.6	6.4	0.3	0.3	0.3
								
**Computation Time (seconds)**								
	***J***	**HC**	**DynTree**	**HOPACH (best)**	**HOPACH (greedy)**	**MM(1–6)**	**RPMM (ICL-BIC)**	**RPMM (BIC)**

**Case 1**	25	0.00	0.04	4.15	1.18	36.39	13.80	13.83
	50	0.01	0.05	3.29	1.09	51.14	14.23	14.23
	500	0.03	0.08	2.98	1.04	436.82	90.99	91.05
	1000	0.06	0.11	3.05	1.10	848.10	176.99	176.81
								
**Case 2**	5	0.00	0.04	2.80	1.21	29.73	5.14	6.09
	10	0.00	0.04	2.01	1.13	46.48	9.69	10.05
	25	0.00	0.01	3.33	1.23	34.56	8.85	8.86
	50	0.01	0.01	2.63	1.16	47.52	10.90	10.86

HC = Hierarchical clustering

DynTree = Hierarchical clustering with classes determined by dynamic tree cutting

HOPACH(best) = HOPACH with 'best' number of classes

HOPACH(greedy) = HOPACH with 'greedy' number of classes

MM(1–6) = Beta mixture model fitting 1–6 classes sequentially

RPMM (ICL-BIC) = Recursively partitioned mixture model employing ICL-BIC

RPMM (BIC) = Recursively partitioned mixture model employing BIC

*J *= Number of loci considered in analysis

**Table 2:** Number of classes obtained for various clustering methods applied to simulated data

	**Case 1 (5 true classes)**				**Case 2 (4 true classes)**			
**Method**	***J***	**Median**	**Mean**	**SD**	***J***	**Median**	**Mean**	**SD**
**DynTree**	25	3	2.5	0.50	25	2	2.0	0.00
	50	3	2.5	0.50	50	2	2.0	0.00
	500	3	2.7	0.58	500	2	2.0	0.00
	1000	3	2.8	0.59	1000	2	2.0	0.00
								
**HOPACH (best)**	25	40	38.0	12.10	5	17	18.9	9.10
	50	35	35.4	11.38	10	14	15.0	8.27
	500	23	23.0	9.52	25	25	24.7	9.80
	1000	23	23.1	9.47	50	25	25.3	7.34
								
**HOPACH (greedy)**	25	8	13.4	14.41	5	5	7.1	6.35
	50	6	11.9	12.66	10	5	7.1	7.11
	500	5	6.6	5.19	25	7.5	10.8	8.52
	1000	4	6.2	4.41	50	8	10.1	7.85
								
**RPMM**	25	8	7.7	2.00	5	2	2.0	0.10
	50	5	5.6	1.32	10	2	2.4	2.28
	500	5	5.0	0.22	25	4	4.0	0.20
	1000	5	5.0	0.00	50	4	4.1	0.58

DynTree = Hierarchical clustering with classes determined by dynamic tree cutting

HOPACH(best) = HOPACH with 'best' number of classes

HOPACH(greedy) = HOPACH with 'greedy' number of classes

RPMM = Recursively partitioned mixture model employing BIC

*J *= Number of loci considered in analysis

In the Methods section we propose a variant of BIC to compare model fit for different numbers *J *of loci. For Case I, the mixture models always minimized the "augmented BIC" at *J *= 1000, while for Case II, the mixture models always minimized the augmented BIC at *J *= 25. For Case I, nearly all 1413 dimensions were at least somewhat informative; it is interesting to note that *J *was always minimized at its highest value for this case. For Case II, the number of informative dimensions was *J *= 20, so the minimum *J *was closest to the true number of informative markers among the *J *considered in this simulation. In additional simulations that used a finer mesh of *J*, *J *was minimized at 20. Similar results were obtained when the classes were less balanced (e.g. Case I with class probabilities respectively 0.15, 0.30, 0.2, 0.25, and 0.1).

### Normal Tissue

**Figure 1:**
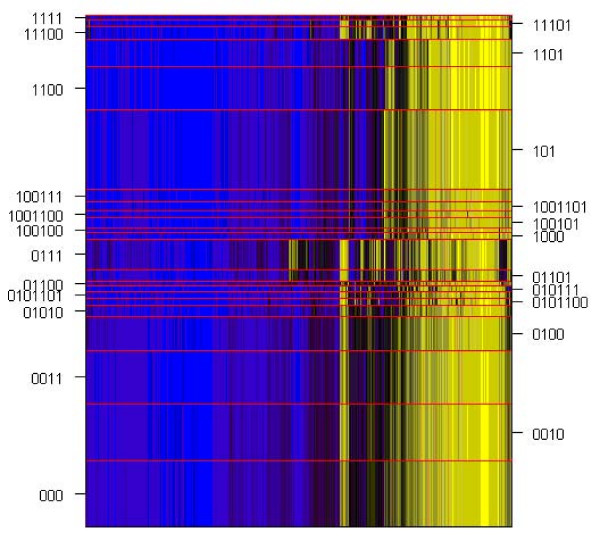
**Profiles of latent classes among normal tissue samples**. Average value (equation 1) depicted by color: yellow = 1.0, black = 0.5, blue = 0.0. Classes are separated by yellow dividing line, with height indicating the relative proportion of subjects within each class. Loci are ordered by their position in a dendrogram obtained via hierarchical clustering.

We applied the recursively-partitioned mixture model algorithm to the normal tissue described below in the Methods section. For this analysis, we attempted to split a node only if the weight assigned to the node was greater than 5. The augBIC_*J *_statistic, which we propose as a comparative measure of model fit for different numbers *J *of loci, was minimized at *J *= 1413, and the algorithm found 23 classes, whose profiles [mean values calculated using (1)] are depicted in Figure 1. All posterior class membership probabilities were indistinguishable from 0 or 1 within numerical error. Table 3 displays the cross-classification between mixture model latent class and tissue sample type. Blood samples were completely separated from other solid tissue samples. In addition, adult blood samples were completely separated from newborn blood samples obtained from Guthrie cards. Placenta samples were also separated from other tissues aside from a single pleura sample. For the most part, head and neck tissue and brain were separated from other samples, but were poorly distinguished between each other. These results were consistent with a Random Forests analysis [20] (8.9% total classification error, additional results not shown), in which we found blood perfectly classified, low classification error for placenta, and some confusion among head and neck tissue and brain tissue.

**Table 3:** Cross-classification of sample type with latent classes obtained from proposed method

Class	bladder	blood (ad)	blood (nb)	brain	cervical	H & N	kidney	lung	placenta	pleura	sm intestine	Total
000	3						2	12		8	3	28
0010								19		5		24
0011								20		2	1	23

0100	2			2	1		4	2		2	1	14
01010				1		4						5
0101100						3						3
0101101						3						3
010111					2							2
01100									1	1		2
01101									5			5
0111									13			13

1000			3									3
100100			2									2
100101			4									4
1001100			3									3
1001101			4									4
100111			5									5
101			34									34

1100		18										18
1101		12										12
11100				5								5
11101				3								3
1111				1		1						2

Total	5	30	55	12	3	11	6	53	19	18	5	217

Classes are labeled with the sequence vector representing the terminal node from which the class was derived.

Using a permutation test with chi-square statistic, the P value for a hypothesis of no association between class and sample type was less than 0.0001. Thus, our proposed method found clusters relevant to sample type. In addition, a permutation test using a Kruskal-Wallis test statistic produced P < 0.0001 for a hypothesis of no difference in mean age among the classes. Interestingly, when the clustering and subsequent hypothesis test was restricted to blood, the P < 0.0001 for a hypothesis of no difference in mean age among latent classes. Between the two classes found among the adult liquid blood samples, age was significantly different (P < 0.0039), consistent with known associations between age and methylation.

Note that the recursion path contains information about relative similarity. For example, in Figure 1, the classes (0...) seem rather distinct from the classes (1...). There is a band of loci that show high methylation in two classes at the top of the figure, indexed as 11101 and 11100, corresponding to brain tissues, but not in the other classes in the left node (1...), mostly corresponding to blood samples; this band also occurs at the bottom of Figure 1, in classes such as 01010, which also include brain tissues. However, as in any hierarchical clustering procedure, the ordering of the children within a node is meaningless, so that classes (11...) and (10...) could have their positions swapped. There is a wider band of loci to the right, which though the distinction may be subtle, appears to drive the distinction between (0...) and (1...). However, because the initial levels of recursion may constitute only a crude splitting of the data, the tree representing the recursion path likely has greater meaning only at lower levels of recursion.

We also analyzed the normal tissue methylation data using HOPACH. The greedy version of the algorithm produced only 4 classes. The "best" version produced 9 clusters, which are cross-classified with tissue type in Table 4 and with the latent classes obtained from our proposed method in Table 5. As Table 5 shows, the classes found by our proposed method were, for the most part, subsets of the 9 classes found using HOPACH, with a few exceptions that involve minor disagreements in classification. While the apparent compactness of the HOPACH classification seems, at first glance, more attractive than the classification produced by our model-based method, we remark that is has a few subtle problems. It has three singletons, clusters 6, 8, and 9, which could be grouped together with cluster 7 to comprise a class that entirely represents placental tissue. While a similar criticism could be made of our proposed method with respect to classification of blood, some of the classes have verifiable meaning; for example, the classes indexed 1100 and 1101 distinguish age among blood samples taken from adults. HOPACH classification also associates one head and neck tissue sample with blood, and two cervical samples with numerous other tissues in the 5^th ^class. The 5^th ^class associates numerous tissue types, and comprises 6 different classes produced by our proposed method. Table 6 shows the cross-classification of tissue type with these 6 classes for the subjects comprising the 5^th ^HOPACH class. The classification correctly isolates two of three cervix samples, and has a tendency to distinguish pleura from lung samples. Using a permutation test with chi-square statistic, the P value for a hypothesis of no association between class and sample type among the 6 classes from our model-based method was less than 0.0001, demonstrating that the classes produced by the mixture model have additional information with respect to tissue type. In order to compare the predictive ability of the two classification schemes overall, we applied the Random Forest algorithm to indicator variables representing HOPACH clusters (using all 9 variables for every bootstrap) and to indicator variables representing our model-based classification (using all 23 variables for every bootstrap). In the former case we obtained a misclassification rate of 18.43%, and in the latter case a misclassification rate of 17.97%, the difference being the misclassification of one less sample using the model-based method. Employing the Random Forest algorithm in a similar manner to predict age, we obtained a mean-squared-residual of 190.1 for HOPACH and 164.8 for the model-based classification, with variance explained equal to 80.6% and 83.2% respectively. Thus, the model-based classification seems to offer modest improvements over HOPACH in ability to make biological distinctions.

**Table 4:** Cross-classification of sample type with clusters obtained from HOPACH

Class	bladder	blood (ad)	blood (nb)	brain	cervical	H & N	kidney	lung	placenta	pleura	sm intestine	Total
1		30				1						31
2			55									55
3				10								10
4				2	1	10						13
5	5				2		6	53		18	5	89
6									1			1
7									16			16
8									1			1
9									1			1

Total	5	30	55	12	3	11	6	53	19	18	5	217

**Table 5:** Cross-classification of latent classes obtained from proposed method with clusters obtained from HOPACH

Class	1	2	3	4	5	6	7	8	9	Total
000					28					28
0010					24					24
0011					23					23

0100			2		12					14
01010				5						5
0101100				3						3
0101101				3						3
010111				1	1					2
01100					1	1				2
01101							4		1	5
0111							12	1		13

1000		3								3
100100		2								2
100101		4								4
1001100		3								3
1001101		4								4
100111		5								5
101		34								34

1100	18									18
1101	12									12
11100			5							5
11101			3							3
1111	1			1						2

Total	31	55	10	13	89	1	16	1	1	217

Rows represent classes from proposed method, labeled with the sequence vector representing the terminal node from which the class was derived. Columns represent clusters from HOPACH.

**Table 6:** Cross-classification of sample type with latent classes obtained from proposed method among subjects within the 5^th ^class obtained by HOPACH

Class	bladder	cervical	kidney	lung	pleura	sm intestine	Total
000	3		2	12	8	3	28
0010				19	5		24
0011				20	2	1	23
0100	2	1	4	2	2	1	12
010111		1					1
01100					1		1

Total	5	2	6	53	18	5	89

Classes are labeled with the sequence vector representing the terminal node from which the class was derived.

## Discussion

Our proposed method is a model-based version of the HOPACH algorithm [16]: we recursively use a beta-mixture model [15] to propose a split of an existing cluster, preserving the split only when it is judged on the basis of BIC to better fit the data. We have found that the *integrated-classification-likelihood *BIC (ICL-BIC) [15] and BIC produce practically identical results; this result is not unexpected, since the two differ only in a term involving entropy and a perfect classification results in zero entropy. A high-dimensional data set will tend to result in approximately perfect classification of most subjects, even though each subject has the opportunity to be portioned out over multiple clusters (a distinction between our method and nonparametric methods such as HOPACH). Consequently, in the deeper levels of recursion, the algorithm will not depend on the distinction between BIC and ICL-BIC. For the earlier recursion stages, we assume that there is a strong preference for splitting, which overcomes the entropy penalty in the ICL-BIC.

Siegmund et al. (2004) argue that model-based clustering is preferred in this context over hierarchical clustering [13], a finding that bears out in our simulations. One reason for the superior performance, at least in a high-dimensional context, is that the metric used to characterize the differences in nonparametric contexts may be relatively insensitive to differences in particular dimensions. This may play a role in the apparent differences in classification of normal tissue between our proposed method and HOPACH.

K-means have been used recently to cluster methylation outcomes [12], though the work of van der Laan and Pollard (2003) seems to suggest that HOPACH may yield results that are superior to K-means. In particular, with K-means it is difficult to know how many classes are inherent in the data without resampling-based methods such as the gap statistic [21], with implications for scalability. Also, the "curse of dimensionality" would tend to degrade the performance of procedures such as K-means when there are a large number of clusters and the observed data is of high dimension. In general, nonparametric methods such as the *fanny *algorithm [17] rely on tuning parameters that are difficult to optimize without resampling. An additional problem with non-parametric procedures is that they typically consider only the first moment (mean) of the underlying distributions, ignoring the second-moment (variance) which for DNA methylation as measured by the GoldenGate assay, may play a critical role in distinguishing tissues.

We propose a dimension-reduction strategy which simply ranks candidate dimensions on the basis of some criterion such as variance, fits the top *J *dimensions in a mixture model, and employs an augmented version of BIC to compare model fit across different values of *J*. This is a departure from the penalized-likelihood methods of the kind described in [22], which would become computationally difficult for truly high-dimensional data. Our approach is similar in spirit to supervised principal components methods such as [23]. Interestingly, for the normal tissue data, all 1413 loci were found to be informative. The implication is that methylation at even the least variable locus, COL6A1_P283_F (a locus on chromosome 21 within a gene that encodes the extracellular matrix component collagen VI), contains information about tissue type. In fact, box-plots showing the distribution of COL6A1_P283_F methylation demonstrate great heterogeneity in apparent distribution by tissue type [see Additional file 1], even though all methylation average beta values were less than 0.05. The Kruskal-Wallis P value for testing the association of COL6A1_P283_F methylation with tissue type was P < 0.0001. While this difference could be a subtle artifact of plate-to-plate variability and heterogeneity in plate sample composition, it also may be that the average beta measured by the GoldenGate assay is in fact an average of methylation status over different cell types: that is, tissue samples may consist of cells having heterogeneous methylation states, with each tissue type having a unique mixture. A separate paper describing analysis of normal tissue in detail is under preparation.

We remark that we did not normalize the different plates used in laboratory analysis, for reasons described below. However, when we used the analytical methods of this paper to classify methylation profiles within plate strata and within tissue type strata, we found much greater variability between tissue types than between plates (results not shown). Our results are consistent with expectations developed from an understanding of the current literature on DNA methylation in normal tissues, though we cannot entirely rule out subtle biases introduced by variation between plates. It may be possible to incorporate plate effects in our proposed methodology by modeling the mean response (e.g., see [22]), but the computational considerations are beyond the scope of the present paper.

## Conclusion

In summary, our method appears to have good properties both with respect to classification error and computation time. It achieves these properties by combining the strengths of model-based and hierarchical methods, navigating the underlying clusters quickly through recursive partitioning, but doing so in a way that makes use of a reasonable probability model. This model is also used to compare different dimensions *J *of input, thus refining the discriminative ability in a scalable manner.

## Methods

### Normal Tissue Data

**Figure 2:**
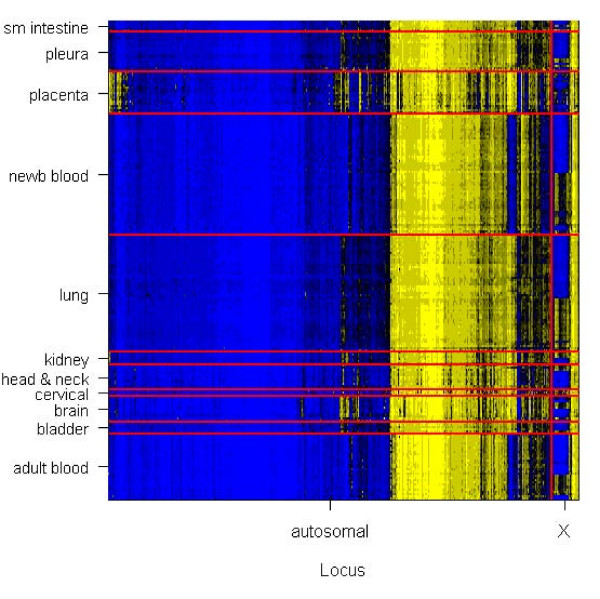
**Unadjusted Average Beta values obtained from Illumina GoldenGate methylation platform for 1413 tumor suppressor loci on 217 normal tissue samples**. Yellow = 1.0, black = 0.5, blue = 0.0. Autosomal chromosomes are grouped to aid visualization. For each chromosome group, loci are ordered by their position in a dendrogram produced by hierarchical clustering. Similarly, within tissue sample groups, samples are ordered by their position in a hierarchical clustering dendrogram.

Our proposed method is motivated by methylation array data obtained for normal tissue. We extracted DNA from 217 normal tissue samples, modified with sodium bisulfite, and processed them on the Illumina GoldenGate methylation platform. Tissues were assembled by a collaborative, multi-institutional network of principal investigators conducting molecular epidemiologic studies of human cancer. Participating institutions include the International Mesothelioma Program at Brigham and Women's Hospital, Brown University, Dartmouth-Hitchcock Medical Center, University of California – San Francisco, Brain Tumor SPORE program, University of Massachusetts – Lowell, and the University of Minnesota. Tissues were obtained through Institutional Review Board approved studies already underway at these institutions, or purchased from the National Disease Research Interchange (NDRI). A variety of normal tissue types were assembled: bladder (*n *= 5), blood (*n *= 85), brain (*n *= 12), cervix (*n *= 3), head and neck (*n *= 11), kidney (*n *= 6), lung (*n *= 53), placenta (*n *= 19), pleura (*n *= 18), and small intestine (*n *= 5). All tissue samples were from adults except *n *= 55 samples of Guthrie card derived blood samples from newborns. Figure 2 illustrates the methylation pattern for all 217 subjects and 1413 loci passing quality-assurance procedures (median detection P-value < 0.05).

These data are part of a larger project comparing normal tissue to tumors for many different types of tumor (e.g. bladder, brain, leukemia, lung, mesothelioma, etc.); each of these comparisons constitutes a distinct set of analyses deserving of a separate, focused analysis, so we omit the details from the present manuscript. In addition, a more comprehensive analysis of normal tissue will appear in a separate manuscript targeted to biologists. Within logistical constraints (e.g. availability of normal tissue, which is difficult to obtain for some tissue types, and time and budget constraints) we randomized tissue type to 14 plates (96 wells each). However, because of the great number of different tissue types, the difficulty in collecting normal tissue of specific types (recall that methylation profiles differ profoundly by tissue type), and heterogeneity of both normal and tumor samples, we expect the plate-to-plate heterogeneity to be dominated the heterogeneity of sample type on each plate. When we used the methods of this paper to classify methylation profiles, stratifying first by plate and examining associations with sample type, then stratifying by distinct tissue-type and examining associations with plate, we found that there are unequivocal differences between sample type on a single plate, and generally no differences between plate within a given sample type, with the possible exception of placenta, whose methylation values have greater variability and therefore may have more sensitivity to random heterogeneity with small sample size. On the other hand, attempts to normalize plates using standard techniques may result in overcorrection, since plate-to-plate variability is mostly driven by sample heterogeneity, and corrections will tend to average out the distinctions of interest.

Note that in addition to providing average "beta" values, the GoldenGate platform also provides averages $\bar{M}$ and $\bar{U}$ (average fluorescence for methylated and unmethylated alleles). For our data, the quantities $\bar{M}/(\bar{U}+\bar{M})$ were close to the reported average "beta" values, with the standard deviation of the difference between the two less than 0.01, a fact which has implications below.

### Recursive-partitioning for a Beta Mixture Model

Under the assumption that $\bar{M}$ and $\bar{U}$ approximately follow gamma distributions on the same scale, the quantity $\bar{M}/(\bar{U}+\bar{M})$, which is close to the reported average "beta" value, follows a beta distribution. In addition, the mean of correlated Bernoulli variables can be modeled by (1), with an appropriate re-parameterization of scale parameters [24]; there is consequently a biological motivation for using a beta distribution. For each single subject *i*, we assume class membership *C*_*i *_∈ {1, ..., *K*}. For methylation data *Y*_*ij *_at locus *j *from subject *i*, we assume the distribution

$$f(Y_{ij}=y|C_{i}=k)=B{\left(\alpha_{kj},\beta_{kj}\right)}^{-1}y^{\alpha_{kj}-1}{\left(1-y\right)}^{\beta_{kj}-1},$$

which depends on both class *k *and locus *j *through the parameters *α*_*kj *_and *β*_*kj*_. Under the assumption that *C*_*i *_= *k *with probability *η*_*k*_, $\sum_{k=1}^{K}\eta_{k}=1$, and that methylation at each locus is independent conditional on class membership, the likelihood contribution from subject *i *is

$$f(Y_{i}=y_{i})=\sum_{k=1}^{K}\eta_{k}\prod_{j=1}^{J}B{\left(\alpha_{kj},\beta_{kj}\right)}^{-1}y_{ij}^{\alpha_{kj}-1}{\left(1-y_{ij}\right)}^{\beta_{kj}-1}.$$

With observed data *D *= {**y**_1_, ..., **y**_*n*_}, we then maximize the full-data log-likelihood,

(2) $$\ell (\alpha ,\beta ,\eta )=\sum_{i=1}^{n}\log\{f(Y_{i}=y_{i})\},$$

with respect to the set of all parameters (**α**, **β**, **η**) to be estimated:

**α **= (*α*_11_, ..., *α*_1*J*_, *α*_21_, ..., *α*_*KJ*_), **β **= (*β*_11_, ..., *β*_1*J*_, *β*_21_, ..., *β*_*KJ*_), and **η **= (*η*_1_, ..., *η*_*K*-1_). This is easily achieved using an Expectation-Maximization (EM) algorithm [25]. Briefly, we initialize the procedure with an *n *× *K *matrix of weights **W **= (*w*_*ik*_) whose rows sum to one. The rows represent initial guesses at class membership probabilities for each subject. For each *k*, we set $\eta_{k}=n^{-1}\sum_{i=1}^{n}w_{ik}$ and maximize the quantity

(3) $$\begin{array}{c} \ell_{k}^{\left(w\right)}=\sum_{i=1}^{n}w_{ik}\log\{f(Y_{i}=y_{i}|C_{i}=k)\}\\ =Q_{k}+\sum_{i=1}^{n}w_{ik}\sum_{j=1}^{J}\left[\alpha_{kj}\log(y_{ij})+\beta_{kj}\log(1-y_{ij})-\log\{B(\alpha_{kj},\beta_{kj})]\}\right], \end{array}$$

where *Q*_*k *_is constant with respect to parameters, to obtain the **α **and **β **parameters corresponding to class *k*. Reversing the order of summation in (3) shows that each dimension *j *can be maximized separately. We subsequently update the weights as follows:

$$w_{ik}=\frac{\eta_{k}\prod_{j=1}^{J}B{\left(\alpha_{kj},\beta_{kj}\right)}^{-1}y_{ij}^{\alpha_{kj}-1}{\left(1-y_{ij}\right)}^{\beta_{kj}-1}}{\sum_{k=1}^{K}\eta_{k}\prod_{j=1}^{J}B{\left(\alpha_{kj},\beta_{kj}\right)}^{-1}y_{ij}^{\alpha_{kj}-1}{\left(1-y_{ij}\right)}^{\beta_{kj}-1}},$$

iterating until ℓ(α, β, *η*) does not change. The final weight *w*_*ik *_represents the posterior probability that subject *i *belongs to class *k*, i.e. *w*_*ik *_= *P*(*C*_*i *_= *k*|**Y**_1_, ..., **Y**_*n*_). As for most finite-mixture methods, we might decide on the number of classes *K *by fitting mixture models for a range of possible values of *K*, computing the BIC statistic

(4) $$BIC=\log(n)(2JK+K-1)-2\sum_{i=1}^{n}\log\{f(Y_{i}=y)\}$$

and selecting the value of *K *corresponding to the minimum BIC. In the context of beta mixture models, a modified version of BIC is available [15]: the ICL-BIC is defined as the sum of (4) and the entropy term $-2\sum_{i=1}^{n}\sum_{k=1}^{K}w_{ik}\log(w_{ik})$ (where 0 log (0) = 0). The entire operation has approximate complexity $nJK_{\max}^{2}$, where *K*_max _is the maximum number of classes attempted. The square term arises under the assumption that for a single model with *K *classes, the complexity will be of order *nJK*.

Because likelihoods for model-based clustering algorithms can be multi-modal [14,26], commercial mixture model software packages often use multiple starting values for fitting the model, and subsequently choose the estimates corresponding to the maximum likelihood. However, careful choice of starting values can often minimize the effort [22,26]. One option is to use hierarchical clustering to find *K *clusters (cutting the clustering dendrogram at the appropriate height), and constructing a weight matrix **W **corresponding to these clusters. Another, similar, option is to use a fuzzy clustering algorithm such as the *fanny *algorithm [17] available in the R package *cluster*.

We now propose a recursive method that, on average, has complexity *nJK*log(*K*), where *K *is the true number of classes. Consider the following weighted-likelihood version of (2),

(5) $$\ell^{\left(\omega \right)}(\alpha ,\beta ,\eta ;\omega )=\sum_{i=1}^{n}\omega_{i}\log\{f(Y_{i}=y_{i})\}.$$

When *ω*_*i *_≡ 1 for all *i*, (2) and (5) are equivalent. When 0 <*ω*_*i *_< 1, subject *i *only partially contributes to estimation, and when *ω*_*i *_= 0, subject *i *is excluded entirely from consideration in the model. The EM algorithm described above is easily modified by multiplying each *w*_*ik *_by *ω*_*i *_in (3), where the interpretation is that the classes under consideration are only a partial set, and that subject *i *belongs to one of these classes only with probability *ω*_*i*_. If we begin by fitting a 2-class model to the entire data set, the result is two sets of posterior weights representing the posterior probabilities of membership in each of the two classes. Under the assumption that each of these classes can be further split, and that each subject belongs to the subsequent splits only with probability equal to the weight assigned to the un-split class, we apply the weighted-likelihood EM algorithm to obtain the two classes corresponding to the new split.

To make this idea more precise, define a concatenation operation *τ *on a sequence of binary values r = (*q*_1_, ..., *q*_*T*_), as *τ*(*r*, *q*) = (*q*_1_, ..., *q*_*T*_, *q*). This provides a natural notation for recursive binary partitioning, where longer sequences represent deeper levels of recursion. The first two-class model, initialized by nonparametric cluster analysis, results in two sets of weights, $\omega_{i}^{\left(0\right)}=w_{i1}$ and $\omega_{i}^{\left(1\right)}=w_{i2}$. For any sequence *r*, a mixture model can be attempted using the weighted EM algorithm with weights $\omega_{i}^{\left(r\right)}$. If the EM algorithm fails, then we terminate the recursion at that point, but if the EM algorithm succeeds, we can set new weights $\omega_{i}^{\left(\tau (r,0)\right)}=\omega_{i}^{\left(r\right)}w_{i1}^{\left(r\right)}$, $\omega_{i}^{\left(\tau (r,1)\right)}=\omega_{i}^{\left(r\right)}w_{i2}^{\left(r\right)}$, and continue the recursion. Note that at each level of recursion, the weights become smaller; since a mixture model becomes unstable with small weights (corresponding to small numbers of pseudo-subjects), the recursion ultimately terminates completely at a set of leaf nodes corresponding to un-split classes. We can stabilize this process by terminating the recursion if the sum of the weights is less than some pre-specified value (e.g. 5). We can also terminate early if the split leads to a less parsimonious representation of the data. To this end, we propose the following weighted versions of BIC:

$$\begin{array}{l} wtdBIC_{2}(r)=(4J+1)\log\left(\sum_{i=1}^{n}\omega_{i}^{r}\right)-2\ell^{\left(\omega \right)}(\alpha^{\left(r\right)},\beta^{\left(r\right)},\eta^{\left(r\right)};\omega^{\left(r\right)}),\\ wtdBIC_{1}(r)=2J\log\left(\sum_{i=1}^{n}\omega_{i}^{r}\right)-2\ell^{\left(\omega \right)}(\alpha^{*(r)},\beta^{*(r)},\eta^{*(r)};\omega^{*(r)}), \end{array}$$

where the first set of parameters, defining wtdBIC_2_, are obtained from the two-class mixture model and the second set of parameters, defining wtdBIC_1_, are obtained from a one-class model. Alternatively, one may add an entropy term to wtdBIC_2_(*r*) to produce the ICL version of this BIC value; note that for a one-class model, entropy is always zero. If wtdBIC_2_(*r*) is greater than wtdBIC_1_(*r*), we terminate the recursion at node *r*. The worst-case complexity of this algorithm is *n*log(*n*)*J*. However, at deeper levels of recursion, two-class models will tend to fit poorly relative to single-class models, and most nodes will terminate before descending to the deepest levels. We have demonstrated empirically that the proposed method tends to terminate with the number of leaf classes equal to the true number of classes, so that the average complexity is typically of approximate order *nJK *log(*K*). Furthermore, in the deeper classes, subjects whose weights are negligible can be dropped entirely from the weighted EM algorithm, so that the complexity of the node-level fit at deeper levels is much less than *n*.

### Dimension reduction

Non-informative loci may lead to excessive noise in the solution. Regularization methods may be used to constrain the degrees-of-freedom, leading to more precise solutions [22,27]. However, in extremely high dimensions, it can also lead to increased computation time and curtail scalability. We propose an alternative, where all *L *starting loci are ordered with respect to variance, and the *J *most variable loci are selected for inclusion in the recursive algorithm described above. A final BIC value can be obtained using (4) by considering all leaf-level un-split classes as distinct clusters, with class prevalence parameter vector **η **obtained by summing the final weights $\omega_{i}^{\left(r\right)}$ and dividing by *n*. However, this BIC is not comparable across different values of *J*. Note that the exclusion of *L *- *J *loci is equivalent to the assumption that all *K *classes have identical distributions for the excluded loci. Thus, beta distributions can be fit to each excluded locus using maximum-likelihood, and the resulting parameter estimates included in a final BIC statistic. Specifically, the likelihood for the full data $Y_{i}^{*}=(Y_{i1},...Y_{iJ},Y_{i(J+1)},...,Y_{iL})=(Y_{i},{\tilde{Y}}_{i})$, where we assume the dimensions have been ordered by descending variance and ${\tilde{Y}}_{i}$ represents data excluded from the mixture model analysis, can be expressed as

$$\begin{array}{c} f^{*}\left(Y_{i}^{*}=y_{i}^{*}\right)=\left\{\sum_{k=1}^{K}\eta_{k}\prod_{j=1}^{J}B{\left(\alpha_{kj},\beta_{kj}\right)}^{-1}y_{ij}^{\alpha_{kj}-1}{\left(1-y_{ij}\right)}^{\beta_{kj}-1}\right\}\\ \times \prod_{l=J+1}^{L}B{\left(\alpha_{l}^{*},\beta_{l}^{*}\right)}^{-1}y_{il}^{\alpha_{l}^{*}-1}{\left(1-y_{il}\right)}^{\beta_{l}^{*}-1}\\ =f\left(Y_{i}=y_{i}\right)\tilde{f}\left({\tilde{Y}}_{i}={\tilde{y}}_{i}\right). \end{array}$$

The "augmented" BIC is now

$$\begin{array}{c} augBIC_{J}=(2JK+K-1+2L-2J)\log(n)-2\sum_{i=1}^{n}\log f(Y_{i}^{*}=y_{i}^{*})\\ =BIC_{J}+2(L-J)\log(n)-2\sum_{i=1}^{n}\log\tilde{f}({\tilde{Y}}_{i}={\tilde{y}}_{i}), \end{array}$$

where BIC_*J *_is the BIC computed for just the *J *selected loci. The augmented BIC is now comparable across different values of *J*. As we have demonstrated, augBIC_*J *_leads to sensible dimension reduction. Again, an ICL version of augBIC_*J *_may be used instead.

### Simulation

**Figure 3:**
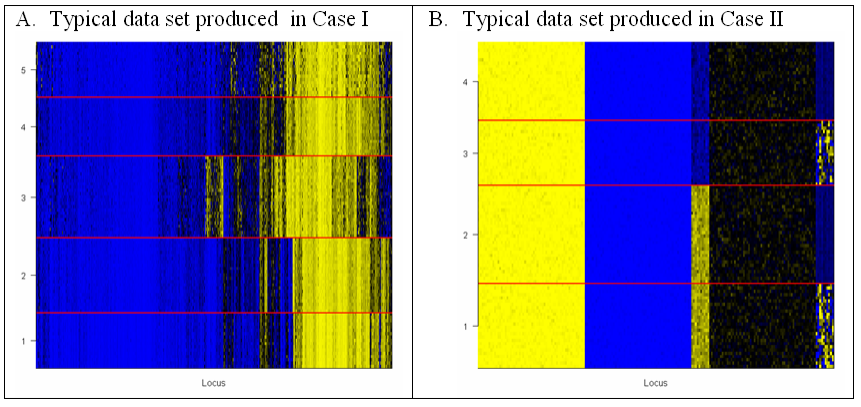
**Examples of simulated data**. Yellow = 1.0, black = 0.5, blue = 0.0. True classes indicated and separated by yellow dividing line. Height of region indicates the relative number of subjects in each class.

We conducted simulations to compare the properties of our proposed method with similar competing methods. For our first case (Case I), each simulated data set consisted of *n *= 100 subjects, each having 1413 continuous responses lying in the unit interval. Each subject was a member of one of 5 classes, each class occurring with 0.2 probability. The classes were defined by beta-distribution parameters for each of *L *= 1413 methylation loci that were autosomal and passed quality-assurance, obtained by fitting a beta model on each locus to one of five data sets from our normal data: adult blood, newborn blood, placenta, lung/pleura, and everything else. Figure 3(A) illustrates a typical data set generated from these parameters. For each data set, we conducted 7 analyses, each using the *J *most variable loci, *J *∈ {25, 50, 500, 1000}. The first analysis used hierarchical clustering, implemented using *hclust *in the R *cluster *package, with Euclidean metric and average linkage, and assigned 5 classes by cutting the resulting dendrogram at the appropriate height using the *cutree *function in the same package. The second analysis used the same clustering procedure, but determined the number of classes using the Dynamic Tree Cutting algorithm [28], implemented via the R package *dynamicTreeCut *with default settings. The third analysis used HOPACH (R *hopach *package) to select the "best" classes as defined in the function settings. The fourth analysis used HOPACH with classes obtained by the "greedy" version of the algorithm. The fifth analysis fit 6 sequential mixture models (1 ≤ *K *≤ 6), each initialized two different ways (hierarchical clustering and the *fanny *algorithm), selecting the value of *K *producing the lowest BIC. The fifth and sixth analyses were applications of our proposed method, using ICL-BIC and BIC respectively. In the last three types of analysis, we recorded the value of *J *that produced the best augmented BIC. Note that each mixture model analysis tended to produce perfect classification, so that an ICL-BIC version of the time-consuming fourth analysis was deemed unnecessary.

For our second case (Case II), which represented a lower-dimensional setting (*L *= 200) with greater variation in variance of individual beta distributions, we considered 100 subjects from 4 classes, described as follows. Four sets of 10 "informative" beta parameters were drawn randomly at the beginning of the simulation study: *a*_1*j *_~ *Gamma*(10,10), *b*_1*j *_~ *Gamma*(10,10); *a*_2*j *_~ *Gamma*(400,10), *b*_2*j *_~ *Gamma*(100,10); *a*_3*j *_~ *Gamma*(100,10), *b*_3*j *_~ *Gamma*(400,10); and *a*_4*j *_~ *Gamma*(100,1), *b*_4*j *_~ *Gamma*(250,1). These were used to construct four classes of 20 informative dimensions: **α**_1 _= (**a**_2_, **a**_1_), **α**_2 _= (**a**_2_, **a**_4_), **α**_1 _= (**a**_3_, **a**_1_), **α**_1 _= (**a**_3_, **a**_4_), *where ***a**_1 _= (*a*_1*j*_), and similarly for the **β**_*k *_parameters with **b**_*l *_= (*b*_*lj*_). Each such 20-dimensional parameter was augmented with a set of 180 "noninformative" parameters, constructed as 60 copies of the vector (100,1,50) for **α**_*k *_and 60 copies of the vector (1,100,50) for **β**_*k*_. The class probabilities were respectively 0.2,0.3,0.2, and 0.3. Although the pattern corresponding to this collection of parameters may be difficult to visualize from the description, Figure 3(B) shows a typical data set generated under these conditions, and reveals a small set of informative markers, some having distinctions in mean and others in variability. Similar analyses were conducted forth is simulation, except with *J *∈ {5, 10, 25, 50}, and 4 classes assumed for hierarchical clustering.

Misclassification rate was assessed for all simulated data sets and analyses. Each estimated class was matched to true class by minimizing the distance between the *J *means calculated according to (1). When the number of estimated classes was greater than the true number, multiple estimated classes were assigned to a single matching true class, thus generating no misclassification error when the estimated class merely partitioned the true class. When the number of estimated classes was fewer than the true number, subjects within true classes that failed to match to an estimated class were considered misclassified. In the latter case, coarsening of the true classes would lead to the smaller absorbed class being judged as misclassified. In the Results section, we showed that HOPACH tends to overestimate the number of classes for the cases we considered, so our strategy, which favors inappropriate partitioning over inappropriate coarsening, is conservative with respect to comparison with HOPACH in this set of simulations.

We conducted additional simulations, for which we omit detailed results. We used normal distributions having the same mean and standard deviation implied by the beta-based simulation described in the text. For assessing classification error, estimated classes were matched to their true classes by matching the fitted alpha and beta parameters to the corresponding parameterization of mean and standard deviation, i.e. inverting (1), in the manner described above. We conducted another simulation based on a different configuration: 200 items, each generated as a random normal with mean and standard deviations as described below, but subsequently transformed as *n*^-1 ^{*r*_*i *_- 0.5}, where *r*_*i *_is the rank of the *i*th value, to obtain a rank-based transformation of the data. The means were generated as 25 random standard normal variables (once at the start of the simulation study) for each of 4 classes, concatenated with 175 zeroes; and the standard deviations were generated as a Gamma(5, 500) (once at the start of the simulation study) for each of 4 classes, concatenated with 175 repeats of 0.01. Note that this configuration represents a substantial departure from the beta distribution, since the marginal distribution of the data are forced to have a uniform distribution. For assessing classification error, estimated classes were matched to their true classes by matching the fitted alpha and beta parameters to the corresponding parameterization of conditional mean and standard deviation, obtained via simulation.

### Validation of normal tissue results by Random Forest

To validate results, we conducted a supervised Random Forest analysis [20] using the R package *randomForest *with 20,000 trees and a value of $38\approx \sqrt{1413}$ (the default) for the *mtry *parameter (number of randomly selected variables per bootstrap). We also tried values of 14 and 76 for *mtry*, obtaining slightly greater classification error. Both tissue type (categorical with 11 levels) and age (continuous) were used as response variables. Detailed discussion of these results will appear in a separate manuscript written for biologists. In addition, we conducted a Random Forest analysis on the classification indices for the 9 HOPACH classes and 23 mixture model classes described in the Results section, with *mtry *= 9 and mtry = 23, respectively, and 20,000 trees.

## Availability

An implementation of the methods described in this paper is available [see Additional file 2]. The R environment is required for the software. Additional supporting functions are available from the authors upon request.

## Authors' contributions

EAH developed statistical methodology, with critical intellectual input from RFY. BCC prepared laboratory samples and coordinated analysis with Illumina; BCC and CJM participated in testing software on methylation data obtained from real tissues. CJM, MRK, MW, HHN, JW, SZ, JKW, and KTK contributed tissue samples. All eleven authors participated in weekly conference calls and discussed the biological implications of the analytical methods.

## Supplementary Material

Additional file 1 Distribution of DNA methylation average beta values by tissue type at least variable locus. Click here for file

Additional file 2 RPMM Software Public Release 1-1. Compressed folder containing R code with examples on simulated data. Click here for file
