# Supplementary figures and images for: Model-based clustering of DNA methylation array data: a recursive-partitioning algorithm for high-dimensional data arising as a mixture of beta distributions
Source: BMC Bioinformatics. 2008 Sep 9;9:365. doi: 10.1186/1471-2105-9-365 (PMC2553421; doi:10.1186/1471-2105-9-365)

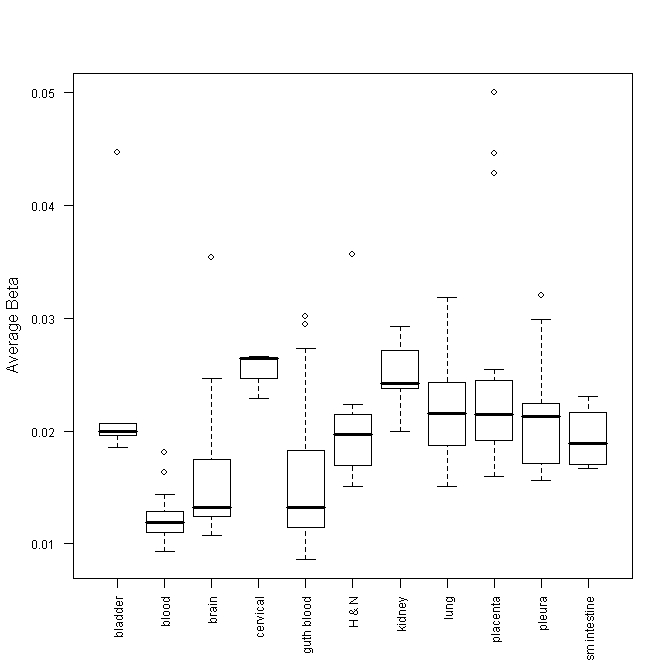

Supplement: Additional file 1 — Distribution of DNA methylation average beta values by tissue type at least variable locus. [file 1471-2105-9-365-S1.jpeg]
